# The Effect of Joint Production on the Accuracy and Complexity of Second Language Writing

**DOI:** 10.1007/s10936-022-09882-8

**Published:** 2022-05-18

**Authors:** Zilin Sang, Weicheng Zou

**Affiliations:** 1 Faculty of Education, East China Normal University, Shanghai, 200063 China; 2 School of Foreign Languages, East China Normal University, Shanghai, China

**Keywords:** Joint production, Corrective feedback, Teacher scaffolding, Collaborative dialogue, Accuracy, Complexity

## Abstract

Although written corrective feedback and collaborative writing have been extensively researched in second language writing, there have been few ecologically valid classroom-based studies. To bridge the gap, the current study proposed joint production as a pedagogy to integrate teacher-scaffolded feedback and collaborative dialogue and aimed to examine its effect on the development of second language writing. A quasi-experimental study was undertaken on two intact groups of EFL learners over an academic semester of 18 weeks. Results showed that the experimental group (N = 30) outperformed the control group (N = 29) significantly in accuracy, confirming the effectiveness of joint production in promoting accuracy in L2 writing. Developmentally, accuracy was steadily developed over time and was negatively correlated with complexity. Possible explanations and implications of the findings are also discussed.

## Introduction

Second language writing (L2 writing) serves the purposes of communicating and learning (Harmer, 2004). However, for L2 writing teachers, the latter seems to weigh more because writing provides a chance for learners to modify their language towards the target and develop their language competence with the assistance of peers or teachers (Qu, 2017). Thus how to promote second language development is invariably a central concern for practitioners of L2 writing. Over time, a wide range of pedagogies have been proposed and implemented to help L2 learners improve their linguistic performance in writing, resulting in a proliferation of studies. It is worth noting, however, that a majority of the proposed practices are based on corrective feedback and collaboration (Bitchener & Ferris, 2012; Bitchener & Storch, 2016; Boggs, 2019; Storch, 2019), seeking to establish the effectiveness of peer and teacher assistance in improving the language quality of learners’ written performance.

Fruitful as they are, however, the existing studies are mostly lab-based and thus lack ecological validity in its design and ultimately, generalizability for different contexts of learning. Considering the complexity of L2 writing classrooms (Ortega, 2009), particularly EFL (English as a foreign language) writing classrooms, more classroom-based practices and research are in need to better accommodate the conditions and needs of EFL learners. The current study proposes joint production as a classroom-based pedagogy on the basis of the existing scholarship in feedback and collaboration and seeks to explore its effect on the accuracy and complexity of EFL writing.

## Literature Review

### Written Corrective Feedback

Defined as responses to linguistic errors in learners’ written output, written corrective feedback is a conventional tool for teachers to improve L2 learners’ language quality and promote their language development and has generated a plethora of studies on the effectiveness of different types of feedback on L2 writing as well as on L2 development (Li & Vuono, 2019). Research interest in feedback is driven by the interaction hypothesis and sociocultural theory in second language acquisition. Interactionists posit that negative evidence, embodied in corrective feedback in negotiations of meaning, can direct learners’ attention from communication to language forms and ultimately their modification of the flawed language. In sociocultural theory, learning is essentially social and occurs within learners’ zone of proximal development (Vygotsky, 1978). Feedback from an “expert” can help the “novice” learner to develop intramentally and self-regulate, and thus promote second language development. In both theoretical lines, feedback serves to raise learners’ awareness and foster their metalinguistic ability.

Extensive research has consistently confirmed that that feedback, either focused or unfocused (Van Beuningen et al., 2012; Lee, 2020), delivered directly or indirectly (Fukuta et al., 2019; Han, 2019), with or without metalinguistic explanation, by the instructor and peers alike (Lundstrom & Baker, 2009), via error correction or tutoring (Han, 2019), takes positive effect on L2 writing, particularly at the revision stage, even though the transferability of the benefit to new texts remains precarious.

Recent scholarship, however, has expanded the research to include more detailed analyses of teacher roles and learner engagement. It is generally agreed upon that teachers should engage in meaning-making, knowledge-co-constructing interactions with the students who actively explore language forms. It is in the process that they provide feedback by scaffolding students’ language production (Carless et al., 2011). Through scaffolding, the teacher and learners jointly participate in the meaning-making processes in which thinking can be externalized and transformed into new knowledge. As proposed by Lee (2017), feedback by this means is descriptive and diagnostic. Clear as the rationale is, how its implementation affects L2 writing remains largely unknown. The existing studies of teacher scaffolding as feedback provision have identified its benefits to L2 writing (Boggs, 2019; Zheng & Yu, 2018). For example, Boggs (2019) compared the effects of teacher-scaffolded (1 to 1 conferences), self-scaffolded, and unscaffolded direct corrective feedback and found that all these led to significant and durable increases in grammatical accuracy in EFL writing. Similarly, Li & Zhang (2021a) confirmed the positive effect of teacher-scaffolded WCF on L2 written language accuracy and rhetorical genre skills. They also delineated and summarized the functions of teacher scaffolding as analyzing language, verbalizing thought, raising awareness, and developing learner autonomy.

The effectiveness of WCF is also mediated by learner factors. Among the factors are proficiency levels, learning goals, learner beliefs, metalinguistic knowledge, and motivation. The benefits of WCF may be proficiency-dependent, with the learners of mid-proficiency level gaining the most in language proficiency and rhetorical skills in EFL writing (Li & Zhang, 2021a, 2021b). Besides, learner engagement with WCF, conceptualized as a process of perceiving and acting upon embedded learning opportunities afforded by WCF, is affected by learner factors such as motivation and metalinguistic knowledge and in turn affects the benefits of WCF (Han, 2019; Han & Hyland, 2015). However, it is also reported that learner perceptions of the types and usefulness of WCF showed no significant difference (Sinha & Nassaji, 2021). This inconsistency should be addressed in future studies.

So far, WCF research has resolved some complicated issues in L2 writing and established some widely accepted criteria for effective feedback. For example, according to Lee (2017), teachers should deliver descriptive, diagnostic information to students about their writing and enable students to close the gap between current and desired performance, helping them improve their future performance, to list a few. In their summary of the criteria for effective feedback, Andrade and Evans (2016) propose that teachers should customize responses to individual learner needs and make responses interactive.

Notwithstanding the improvements, there still leaves much to be desired when it comes to L2 writing pedagogies in the classroom. Firstly, even though some researchers have pointed out the importance of ecological validity in the design of studies (Han, 2019; Lee, 2020; Li & Zhang, 2021a, 2021b), there is an obvious need for more longitudinal ecologically valid classroom-based studies (Storch, 2018). Secondly, error correction, as is typically practiced, takes the form of submission-correction-revision in L2 writing classes. Such procedures might be inherently flawed in that feedback delivery lags far behind learners’ composing, thus likely to mitigate the potential effect of the treatment on both revision and new text writing (Evans et al., 2010). To date, most researchers and teachers agree that corrective feedback on L2 writing takes maximal effect when it is situated, meaningful, and timely. Therefore, how to contextualize instantaneous corrective feedback within a meaningful context in L2 writing classrooms becomes a key issue to address. Thirdly, most feedback research in L2 writing concentrates on its effect on accuracy development with scant attention spared for its effect on other dimensions of writing such as grammatical complexity or lexical complexity. We believe that teacher feedback should not just be confined to correcting errors. Teachers have a broader role to play in promoting L2 writing development. Therefore, it seems reasonable to include complexity as an indicator of the feedback effects.

### Collaborative Writing

Defined as “an activity that requires the co-authors to be involved in all stages of the writing process, sharing the responsibility for and the ownership of the entire text produced” (Storch, 2019: 40), collaborative writing has been widely practised in L2 writing classrooms and extensively researched. Similar to written corrective feedback, collaborative writing is theoretically motivated by the interaction hypothesis and sociocultural theory (Li & Zhang, 2021b). It is hypothesized that the collaboration and scaffolding in the interactions during collaborative writing tasks are essential to the simultaneous cognitive development and language development. In the L2 writing class, collaborative writing is usually implemented by encouraging learners to exchange ideas, to co-construct texts, and to negotiate for meaning and forms dialogically. In the process of collaborative writing, the need to reach agreement on what to express and how to do it in the jointly produced text encourages learners to deliberate about language choice and grammatical accuracy (Storch, 2019). This is a process of languaging (Swain, 2006) when learners can attend to language forms, experiment with new forms, and provide timely mutual help to bridge the gap between their current proficiency and the desired state. Learners can also seek support from external sources and mutual assistance (Yang, 2014; Li & Kim, 2016; McDonough et al., 2018a, 2018b). Overall, collaborative writing not only allows learners to exchange ideas and enrich the meaning of the text but also creates opportunities for them to scaffold each other in terms of language use. The attention to language is likely to drive second language development and improve language quality in L2 writing. Besides, collaborative language production can prompt learners to deepen their awareness of linguistic rules and trigger cognitive processes that might generate new linguistic knowledge and consolidate existing knowledge. In the meanwhile, metatalk can help learners understand the relation between form and meaning, and positively affect acquisition of L2 knowledge (McDonough et al., 2016). This perspective has been echoed by many researchers and teachers and invited a number of studies.

Empirical research seeks to investigate the effect of collaborative writing by examining the outcomes of collaboration in terms of the quality of the text produced and evidence of language learning and the process of collaborating. Most studies adopt a product-oriented research design (Elabdali, 2021) by comparing collaboratively produced texts and individually written ones in terms of language quality and other indicators (such as authorial voice). A meta-analysis of 33 studies suggests that collaboratively written texts were more accurate than individually written texts, with a mean effect size of a medium magnitude (g = 0.73) and that a large magnitude difference in rubric scores (g = 0.94) was found in favor of individual texts written after experimental CW conditions compared to those written after control individual writing conditions (Elabdali, 2021). For example, Zabihi and Bayan (2020) examined the effect of peer collaboration on an argumentative writing task in the CAF framework (complexity, accuracy, and fluency). Complexity was measured as the number of subordinate clauses to T-units, accuracy as the number of error-free T-units against the total number of T-units, and fluency as the average number of words per T-unit. It was found that pairs outperformed independent writers in all CAF measures, again proving the effectiveness of collaborative writing.

In contrast to these positive findings, several recent studies have generated mixed findings. For example, Vorobel and Kim (2017) reported that though collaborative writing in face-to-face and online contexts benefited English as second language (ESL) learners in terms of improvement of L2 writing and development of communication skills, it also brought them challenges. ESL learners in this study found it difficult to co-construct knowledge if both of them had gaps in their language proficiency and to incorporate feedback from peers. Besides, it was also found that collaborative writing only had significant effect on content, organization, and vocabulary, but not on grammar and mechanics (Sajedi, 2014; Shehadeh, 2011). Shehadeh (2011) attributed the absence of significant effect on grammar and mechanics to the low proficiency level of the participants who were not able to provide feedback to each other. Similarly, a moderate relationship between prewriting collaborative dialogues and the written texts was also reported (Neumann & McDonough, 2015). It is noteworthy that the number of participants constitutes a concern for research on collaborative writing. Dobao (2012) found out that group written work was significantly more accurate than both individual work and pair work. All these mixed findings point to the necessity to further studies of collaborative writing, particularly in the EFL context where writing serves a learning purpose.

On the whole, research so far has been fruitful in that we now have an improved understanding of the relationship between learner interactions and text quality, the nature of group/pair work in collaborative writing tasks, and the effect of collaborative writing on different dimensions of L2 writing (Shehadeh, 2011; Storch, 2019). However, from both theoretical and pedagogical perspectives, research on collaborative writing still needs refining before more robust conclusions can be drawn and applied to L2 writing class. Firstly, most studies adopt a cross-sectional design. Though it can capture the difference at a fixed moment, it may fail to track the development of L2 writing under the influence of collaborative writing. Thus, such a design likely compromises the robustness of the findings as well as their generalizability. Secondly, even though a positive effect of collaborative writing on L2 writing has been confirmed by most studies, its eventual effect on L2 writing quality is not clear yet. In other words, whether the acquired advantage of collaborative writing can be transferred to new text writing remains a moot point. Therefore, there is a need to examine and establish the long-term effect of collaboration on L2 writing development in a refined design. Thirdly, there are also practical considerations before it can be adopted in L2 writing class. As class time is limited, full-length collaborative L2 writing might have to be adapted to better fit in. In the meanwhile, collaborative writing, as reported in most studies, takes place among peers, leaving little role for the instructor to play. Nevertheless, the inclusion of the instructor as a collaborator might boost the effectiveness of collaborative writing. Last but not the least, as collaboration in the form of writing is time-costly, it might be more efficient to encourage learners to jointly produce an oral “written” task. Keeping the essence of collaborative writing while saving class time, such a joint-production approach is predicted to take a positive effect on the language quality of L2 writing. This is a fundamental hypothesis of the current study.

### The Study

#### Research Questions

The study aims to answer the following questions:

1. Does joint production take effect on EFL writing in terms of accuracy and complexity?
2. How do learners perceive joint production as a pedagogy?

## Methods

### Participants

The current study was classroom-based and quasi-experimental. Two intact classes of a similar English proficiency level were recruited from the first-year students of a Chinese university, with the experimental class (N = 30) receiving joint production and the control class (N = 29) doing the same reading and writing tasks. All the participants, aged 18 on average, had been learning English for approximately 10–12 years and were considered lower-intermediate to intermediate in terms of English proficiency. When the study was conducted, they were taking a compulsory reading course taught by the same instructor. None of the students had extended overseas experiences. There were 17 female students in the experimental class and 15 in the control class. A placement test consisting of listening, reading, writing items was administered one week before the study started. The results showed no significant differences between the two classes in English proficiency (t = 0.034, *p* > 0.05).

### Treatment

Informed by research findings in corrective feedback and collaborative writing as well as knowledge of the learning context in question, joint production has been regularly practised by both authors in their EFL classes. It is defined and operationalized as a collaborative post-reading activity featuring peer interactions in collaborative dialogues and teacher scaffolding. The implementation of a joint production activity usually takes approximately 35–40 min and involves 5 stages which are presented in Table 1.

**Table 1:** Procedures of joint production

Stages	Activity
1	Students work individually on the assigned reading material (mostly short stories or essays of about 1,000 words) before class
2	In class, the instructor gives a synopsis task with clear instructions. In case that the reading material defies an easy summary, a few comprehension questions are presented to facilitate their work
3	Students work in small groups on the task. They are encouraged to share ideas and co-construct a text. In case they have questions regarding comprehension or language use, they are allowed to ask the instructor for help or consult a dictionary
4	Students are randomly selected from different groups to report their synopsis verbally with the help of the instructor or to write it down on the board. For oral work, the instructor takes it down verbatim on the board
5	The instructor leads the whole class to correct, polish, or supplement the synopsis
6	The instructor does explicit teaching on the final version of the synopsis

Apparently, joint production centers on language forms in the process of meaning-making. Embedded in it are peer collaboration, corrective feedback, and teacher scaffolding which are believed to be conducive to second language development. Besides, this activity is reading-based, with an emphasis on reading-writing connections, allowing learners to spare less cognitive effort for content and more attentional resources for language forms. Intuitively sound as it is, joint production calls for evidence to prove its efficacy on EFL learners’ second language writing, particularly on the accuracy and complexity of their language in L2 writing.

Both classes were taking the same reading course taught by the same instructor. While joint production was delivered 14 times in the experimental class, regular reading-based activities were undertaken in the control class such as comprehension check, language analysis, and open-ended discussions. Both classes received the same assignments and teacher feedback.

### Data Collection

To track changes in learners’ writing, three writing tasks were assigned to both classes and collected in week 2, week 9, and week 18. In these tasks, students were required to write an essay on topics of wide concern in China (i.e. extension of retirement age) in no less than 200 words. The topics were unrelated to the reading materials and were prejudged by a group of students (N = 28) of similar English proficiency to be equally familiar to college students. Students submitted their writings as part of the requirements for the course credits. Therefore, three texts were obtained for each participant.

In addition, an interview protocol (see “Appendix 1”) was also developed with ten open-ended questions about learners’ attitudes towards joint production as a classroom activity, their perception of the change of their writing ability, and their writing strategies. The interview was conducted independently to ten students of different proficiency levels from the experimental class in week 19 and audio-recorded. All the students volunteered to attend the interview. The recording was approximately 100 min long.

### Data Analysis

Data analysis underwent two stages. Stage 1 involved the quantitative analysis of the writing data. We identified and coded all the errors and T-units in students’ essays by following the guidelines of Polio (1997). All grammatical and lexical errors, and T-units were counted. A T-unit was defined as an independent clause and all its dependent clauses. 10% of the essays were jointly coded by the two researchers, with the inter-rater reliability at α = 0.983 for error identification and α = 0.991 for T-unit counting. The rest of the essays were coded independently by the first author.

As the writing tasks were untimed, our measurement focused on two dimensions of second language writing: accuracy and syntactic complexity. Accuracy was defined as the extent to which learner language conforms to the established rules of grammar and word use in the target language (Ellis & Barkhuizen, 2005) and thus measured as the number of errors per T-unit (E/T) or the number of error-free T-units per T-unit (EFT/T). Complexity, on the other hand, indicates the internalization of new L2 elements (Housen et al., 2012) and is usually operationalized as the degree of clause embedding. It was therefore measured as the number of clauses per T-unit (C/T) and the number of dependent clauses per clause (DC/C) in the current study. The data for syntactic complexity were automatically analyzed by the syntactic complexity analyzer developed by Xiaofei Lu and available at http://www.personal.psu.edu/xxl13/downloads/l2sca.html. The reliability of this tool was confirmed in Lu (Lu, 2010). Definitions and measures of accuracy and syntactic complexity are presented in Table 2 below.

**Table 2:** Linguistic measures of accuracy and complexity

No.	Construct	Definition	Measures
1	Complexity	The number of clauses per T-unit	C/T
		The number of dependent clauses per clause	DC/C
2	Accuracy	The number of error-free T-units per T-unit	EFT/T
		The number of errors per T-unit	E/T

Quantitative analysis underwent two steps. Firstly, descriptive statistics of accuracy and complexity for both classes were presented, involving 3 essays for each participant. The three essays were considered Pre-treatment task, While-treatment task and Post-treatment task respectively. Then a repeated-measures ANOVA was applied to determine the effectiveness of the treatment and the characteristics of second language writing development as a response to the treatment.

Stage 2 dealt with the interview data which were transcribed before being processed manually. After carefully reading the transcriptions and independently coding where learners commented on their attitudes and perception of changes in writing and writing strategies, both researchers resolved the disputes by discussing and established the final themes.

## Results

1. The effect of joint production on accuracy and complexity of L2 writing

The means and standard deviations for different measures of accuracy and complexity of both classes are presented in Table 3. Overall, the two classes exhibited differences in the development of accuracy and complexity over time.

**Table 3:** Descriptive statistics of accuracy and complexity

Construct	Measure	Class	Text 1		Text 2		Text 3	
			M	SD	M	SD	M	SD
Accuracy	EFT/T	Exp	0.45	0.12	0.54	0.11	0.66	0.07
		Ctrl	0.41	0.14	0.50	0.13	0.46	0.13
	E/T	Exp	0.80	0.32	0.64	0.22	0.54	0.21
		Ctrl	0.91	0.29	0.79	0.30	0.84	0.27
Complexity	DC/C	Exp	0.38	0.13	0.42	0.14	0.43	0.14
		Ctrl	0.45	0.12	0.42	0.11	0.41	0.11
	C/T	Exp	1.97	0.80	2.27	1.21	2.07	0.86
		Ctrl	2.44	1.28	2.06	1.04	1.86	0.48

In terms of accuracy, starting at a similar level, both classes found improvement in both EFT/T and E/T over the 18-week period. Nevertheless, it seems that they made progress to different degrees. As joint production was regularly delivered, a gap emerged between the two classes and gradually widened, indicating that the experimental class improved accuracy to a greater extent than the control class. This finding was confirmed by a repeated-measures ANOVA which showed a significant difference between the classes in both EFT/T (F(1,57) = 14.26, *p* < 0.05, η^2^ = 0.20) and E/T (F(1,57) = 9.86, *p* < 0.05, η^2^ = 0.15).

**Fig. 1:**
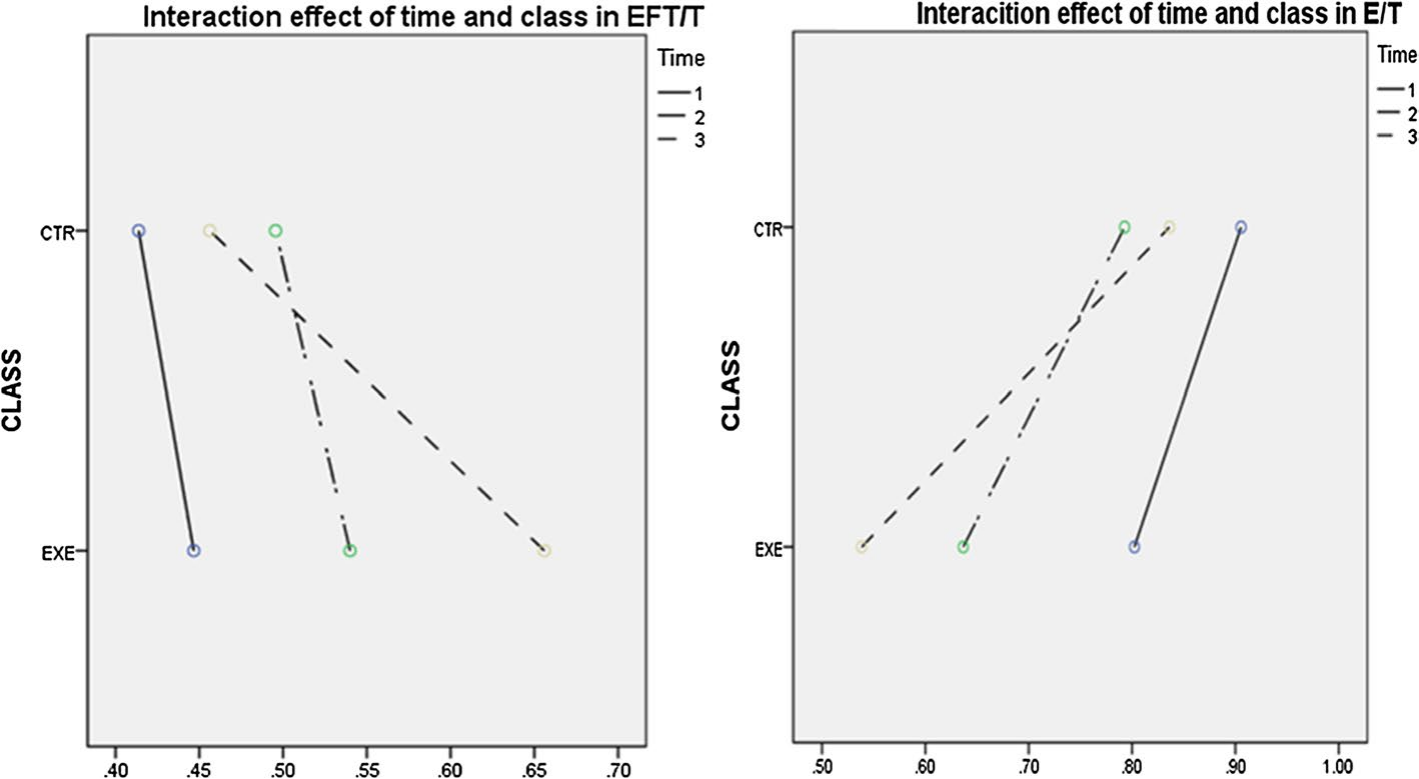
The interaction effect of time and class in accuracy

Besides, there was also a significant interaction effect between time and class for EFT/T (F(2,114) = 15.84, *p* < 0.05, η^2^ = 0.22) and ET/T (F(2,114) = 4.55, *p* < 0.05, η^2^ = 0.16) (see Fig. 1), suggesting that the two groups of EFL writers might have displayed distinct patterns of development in accuracy. This interaction effect, together with the main effect of class, suggests that the experimental class improved accuracy at a significantly higher rate and more consistently than the control class as a result of receiving joint production. In other words, the pedagogical treatment was effective in promoting accuracy of L2 writing.

In contrast, complexity development was characterized by inconsistency and fluctuations for both groups of learners. As shown in Table 3, both measures changed mildly over time in both groups. In the experimental class, subsequent to the delivery of joint production, there was noticeable improvement in both DC/C and C/T from text 1 to text 2, but virtually no progress from text 2 to text 3. The inconsistency was even more apparent in the control class where DC/C regressed from text 1 straight to text 3. These findings seem to suggest that the two classes might not differ much in terms of complexity development. A repeated-measures ANOVA showed that there was no significant difference between classes either for DC/C (F(1,57) = 0.73, *p* > 0.05) or for C/T(F(1,57) = 0.00, *p* > 0.05). Thus it suffices to argue that joint production contributed insignificantly to the development of complexity in second language writing.

With regard to the route of second language writing development within the experimental class, a repeated-measures ANOVA generated a significant time effect for EFT/T (F(2,58) = 51.09, *p* < 0.05, η^2^ = 0.64) and E/T (F(2,58) = 19.39, *p* < 0.05, η^2^ = 0.40). Besides, pairwise comparisons showed significant differences from time 1 to time 3 consecutively for both measures. These results suggest that accuracy in second language writing was steadily improved. In contrast, no significant time effect was found either for DC/C (F(2,58) = 1.668, *p* > 0.05) or for C/T (F(2,58) = 1.449, *p* > 0.05), indicating that complexity in learners’ essays remained stagnant.

In order to determine whether there was a potential relationship between complexity and accuracy, we correlated these two constructs. The results (Table 4) are mixed: if measured as C/T, complexity showed a significant negative correlation with accuracy; if measured as DC/C, it was still negatively correlated with both measures of accuracy, but not at the significance level of 0.05. There seems to be an inverse relationship between accuracy and complexity in the development of L2 writing.

2. Learner perceptions of joint production

**Table 4:** Correlations between accuracy and complexity

	C/T	DC/C
EFT/T	− 0.214*	− 0.107
E/T	0.209*	0.162

*p < 0.05, two-tailed

The interview data generated several themes regarding the effects of joint production on learners. Firstly, most interviewees embrace the activity positively and preferred it to other regularly practised reading-based activities. Secondly, the intensive delivery of feedback from both peers and teachers rendered them sensitive to language forms in the composing process. As a consequence, they adopted writing strategies to monitor their language in the process.

When it comes to learner perceptions of joint production as a classroom activity, most of them responded positively and appreciated the underlying teaching philosophy. When asked about their activities in pair-work discussions, most learners reported that they usually engaged in exchanges of ideas and negotiations for the best language forms to embody their meanings. One learner said:

Generally, we share our understanding of the article and try to reach agreement. When my partner takes the turn to speak, I usually check whether I can understand her. In the meanwhile, I also pay attention to her expressions, especially those I’m not familiar with. When I find some language expressions problematic, I usually point them out. My partner usually does the same.

Another learner supplemented:

To be frank, I am not good at English, so I try to learn from others in the team. He can use many high-end words and sentence structures. While he is speaking, I listen carefully and sometimes take notes. When I speak, he usually helps me correct and improve my language. This is what I appreciate most.

Apparently, the dynamic in peer interactions embodies opportunities for learning: each student can be an expert to the others. Assistance, whether explicitly or implicated stated, is a driving force for learner development in language use.

However, their communication is not without problems. One student said:

Sometimes, I don’t accept my partner’s choice of words, but she insists. So we don’t know who is correct. It is puzzling.

This remark identified one problem with student-led classroom activities: they may be short of necessary expressions to engage in meaning-making. This embarrassing situation can only be resolved by the presence of a teacher or a student of a higher language proficiency. In the observed classes, the teacher usually came to the aid of the students by providing feedback on their language.

Such efforts on the part of the teacher were also felt by learners, as reported by one learner.

I always feel that our language does not fully express our mind. So every time our teacher changes our language, I pay close attention to it. I sometimes take down those expressions I feel useful. I also take pictures when the teacher’s final version is demonstrated.

As a result of the timely feedback provided in their ZPD, students learn quickly. They likely become more form-conscious and better able to control their language production. Therefore, improved language quality should come as no surprise in the follow-up stage of independent writing.

One distinct feature of joint production lies in the cross-modality presentation of WCF which is materialized in written form on the board. In the interview, most students reportedly noticed this and one of them remarked,

Oral feedback is fleeting and hard to get. But I usually have a deeper impression upon the feedback written on the board. I even take notes.

This remark clearly shows students’ preference for materialized written form of WCF which seems more easily understandable and more likely internalized.

When asked to comment on their perceived change in English writing as well as their own writing ability, one of them said,

Back in high school years, I rarely revised or proofread after writing an English composition. But it is different now. I don’t pay much attention to accuracy while composing. But I do spend a lot of time correcting mistakes with lexical use or grammar rules after I complete the first draft. I guess this helps me much.

Another added by telling her strategy to safeguard accuracy in the process of writing:

In order to be accurate, I often consult dictionaries…in most cases, online search engines like Baidu…Besides, every time I finish the first draft, I usually read it several times to eliminate possible mistakes…

These comments show that joint production worked effectively not only on specific language points but also on learners’ developing writing strategies. Chances are that both will contribute to the long-term effect on their written accuracy.

The final question addresses students’ perception of any noticeable change in their understanding of good writing as a result of taking the course. One students commented,

Now I don’t think long sentences reflect one’s English proficiency. In fact, it is easier to write long sentences with a number of clauses embedded as long as you are familiar with English grammar. Because of this, I sometimes intentionally avoid writing long sentences with complex structures. And I am trying to replace some clauses with noun phrases, which I find way harder……

The learner clearly expressed her changing perception of linguistic complexity, which might account for the absence of significant improvement in this criterion. Though measured as a stable linguistic construct, complexity is perceived differently by learners at different proficiency levels. The change in learner perceptions will probably in turn lead to changes in their writing performance. Learners in the current study are at the transitional stage from the intermediate to the advanced level, and therefore likely to develop different understandings of what counts as complex structures.

## Discussion

The current study aimed to examine the efficacy of joint production as a pedagogical treatment in the EFL context and learner perceptions of the activity. It was found out that joint production was effective in developing learners’ writing accuracy, but not in syntactic complexity. This finding might suggest that joint production placed learners at an advantageous position in developing accuracy at a higher rate than their counterparts receiving regular comprehension-based instruction, and prioritized accuracy over complexity. Developmentally, accuracy was steadily improved over time and was negatively correlated with complexity. It indicates that aspects of second language writing might not develop simultaneously at the same pace. In addition, joint production was positively reviewed by students who reported gains from the activity and gradually developed better writing strategies.

Not surprisingly, the finding about accuracy corroborates most recent studies on collaborative writing (Dobao, 2012; Elabdali, 2021; Shehadeh, 2011; Zabihi & Bayan, 2020) and WCF (Van Beuningen et al., 2012; Kurzer, 2018; Fukuta et al., 2019; Han, 2019; Lee, 2020; ; Sinha & Nassaji, 2021), thus further extending and establishing the positive roles of feedback and collaboration in promoting L2 writing development. Similarly, the finding about learners’ lack of development in complexity was also reported in the literature (Dobao, 2012; Sajedi, 2014; Storch, 2005). However, this finding was notably different from Benevento and Storch (2011) which reported increased complexity but unimproved accuracy in the writing of secondary school learners of French. Similarly, Elabdali (2021) found that collaboratively produced texts outperformed independent texts in both accuracy and complexity. Possible explanations for these findings can be found in the inherent features of joint production and learners’ perceptions of this pedagogy and their own changes in writing performance.

As a reliable criterion of the quality of EFL writing and the most straightforward and internally consistent construct, accuracy refers to the extent to which learners’ performance as well as the underlying L2 system conforms to the rules of the target language (Ellis & Barkhuizen, 2005). It incorporates both appropriateness and acceptability. For intermediate learners, maintaining accuracy in writing calls for adequate attention to language forms and the ability to control language and related metacognitive strategies. These needs, however, are accommodated in joint production which integrates teacher scaffolded feedback and collaborative dialogue, two components widely considered helpful in promoting L2 development.

A prominent feature of joint production as a classroom activity is its full-range collaboration between peers and between learners and the instructor. From the sociocultural perspective of learning (Lantolf & Poehner, 2014; Storch, 2018), collaboration encourages learners to scaffold each other in choosing what meanings to convey and what forms to embody meanings, particularly when collaborative dynamics allow for the transfer of knowledge among the group members. In the process of collaboration, learner interactions are usually characterized by the emergence of many language-related episodes (LRE) where they notice, negotiate, correct, and experiment with language forms. As reported by the students interviewed, they did focus on language forms in peer interactions and teacher-student interactions. The analysis of LREs shows that learners are able to resolve some grammatical and lexical difficulties and retain the benefits. Learners in these processes are “individually novices and collectively experts” (Donato, 1994) and thus can scaffold each other in language use. In this way, collaborative dialogues become a platform breeding increasingly accurate language. In the long run, this ability is predictably internalized and transferred to independent writing.

Another feature of joint production is teacher-scaffolded feedback whose provision is dynamic, responsive and individualized (Carless et al., 2011; Kurzer, 2018). It shifts from teaching knowledge to engaging students in meaning making and problem solving in the process of which students can gradually develop their ability to regulate their thinking and language. Besides, teacher-scaffolded feedback is graduated and contingent. Through meaningful interactions, a moderate amount of needs-based feedback is provided, which maximizes the possibility of learners’ internalization of related language points, leading to improved accuracy.

Teacher-scaffolded WCF is comprehensive and targets all language deviations in learners’ production, lexical and syntactic alike. As the production is orally presented, particular attention is paid to the drop of those communicatively redundant markers such as past tense “ed” and plural “-s” forms. The finding that joint production helped improve language accuracy in L2 writing corroborates studies of comprehensive error correction (Van Beuningen et al., 2012) and further confirms its importance in teaching L2 writing.

Error correction, as practiced in joint production, is locally situated, immediate, and itemized (Evans et al., 2011), thus likely to avoid the shortcomings of regular written corrective feedback and optimize its effectiveness. These features guarantee learners’ raised attention to language forms, noticing the gap between their production and the target one, and more importantly, enhanced error correction strategies. Thus trained, learners will likely transfer the acquired abilities to the composing process where they are enabled to apply error-correction strategies and better control their language production. The ultimate outcome is conceivably improved accuracy. An episode of joint production serves as evidence to the point.

While one student was speaking, the instructor took down his words verbatim.

T: (speaking while writing) It’s about a young man called Vingo. He had gone to the jail for four years.

(Repeat it.) Is it possible to combine these two sentences?

S: It’s about a young man called Vingo who had gone to the jail for four years.

T: (Repeat the sentence with a STRESS on “gone”.)

S: (After a few seconds’ silence) been? (very hesitantly)

T: Yeah. It should be “been” instead of “gone”. (repeat the sentence)

……

T: His wife forgived Vingo. (with STRESS on “forgived”)

S: His wife forgiven Vingo.

(Some students were murmuring “forgave”.)

T: Forgiven?

S: His wife forgave Vingo.

In this episode, the learner was orally constructing a synopsis of an assigned reading while the instructor took down verbatim his words on the board and provided immediate feedback on her language. The instructor did so mostly by raising the learner’s attention and eliciting reformulations. The delivery of feedback was instant and situated in communicative activities, thus maximizing the possibility of learners’ noticing and subsequent internalization of the feedback (Andrade & Evans, 2016; Kurzer, 2018; Lee, 2017). More importantly, learners could develop an enhanced awareness of error correction as a learning strategy so that it could be employed in composing new texts. Besides, different from lab studies, classroom studies allow the treatment to be repeated. In this study, joint production was delivered many times and thus helped consolidate learners’ gained abilities and strategies. This feature guarantees the durable effect of joint production on accuracy.

As a matter of fact, teacher involvement in the form of scaffolding is manifested conspicuously at the third and fourth stages of joint production where the instructor joins learners to construct an informationally richer and linguistically better text. In terms of language quality, the instructor makes changes in learner production so that it becomes linguistically accurate and complex. The episode below can illustrate it.

T: What is the article about?

S: It is about the change of the relationship between the writer and his dad.

T: That’s right. (while taking it down on the board) But has everything changed between them?

S: (puzzled and hesitant) …

T: Do they still love each other when their relationship changes?

S: Oh, no. Their love does not change.

T: So you mean “the article is about the changing relationship and the unchanging love between the writer and his dad’?

S: Yes. (nodding)

As part of the interaction, the instructor scaffolded the learner’s production by providing feedback on her language, supplementing information, polishing her language, and negotiating in the whole process. As the interactions are directed to the whole class, students are exposed to the negotiation and scaffolded to independently develop a synopsis.

Learner perceptions also lend support to the above observations. All the students interviewed, though differing in writing abilities, reported improvement in accuracy in their writing. With regard to the “how” and “why” of their progress, most of them attributed much of it to the instruction they had received. To them, joint production worked effectively on their developing ability to write accurately for two reasons. Firstly, they developed a habit of proofreading and revising as a result of joint production which impressed upon them the importance of error correction to L2 writing quality and boosted their awareness and attention to language forms in English writing. Students gradually came to realize that writing was more about revising than drafting.

It is also noteworthy that joint production is characterized by the integration of oral feedback and written feedback. Compared with the traditional error correction strategies, this integrated approach is more likely to focus learner attention to ill-formed language expressions because it weds the advantages of the two types of corrective feedback (Weissberg, 2005). It is timely on one hand and explicit and stable on the other.

Learners’ preference for such feedback seems to suggest that learners’ reception of feedback is as important as its delivery (Han, 2019; Zheng & Yu, 2018). In other words, joint production as an integrated approach of feedback delivery facilitates learners’ reception and subsequent internalization of feedback which in turn drives the development of accuracy in L2 writing.

To summarize, joint production exerts an immediate and lasting effect upon learners primarily in terms of their raised attention to language forms and their improved strategies to self-correct possible mistake in their writing. Therefore, enhanced accuracy emerges in L2 writing.

Developmentally, accuracy and complexity in second language writing followed different trajectories in the current study. In contrast to the virtually linear development of accuracy, complexity, measured as DC/C and C/T, seemed stagnant. Learners did not write structurally more complex sentences in response to joint production for an academic semester. To account for this surprising finding requires an examination of learners’ perceptions of both the classroom activity and complexity in their writings as well as the intricate relationship between accuracy and complexity.

Though notoriously difficult to define, complexity is commonly referred to as the extent to which learners produce elaborated language. There are two layers of senses with “elaborated” language: on one hand, learners vary in their willingness to use more challenging and difficult language; on the other, it also implies learners’ preparedness to use a wide range of complex structures (Ellis & Barkhuizen, 2005). In other words, complexity development is not only determined by learners’ linguistic ability; it also depends on learners’ willingness or perception of the importance of being complex.

One possible reason for the absence of any significant change in complexity resides in joint production itself. Generally speaking, learners complexify sentence structures when they have newly learned a complex structure and become linguistically ready to embed clauses or when they find it necessary to embody and convey elaborate and sophisticated information in complicated structures. However, learners in the current study are all at the intermediate level of proficiency and have no urgent need to experiment with those complex sentence structures acquired long before. In the meanwhile, synopsis writing, the focus task of joint production, requires compression of information rather than elaboration. In other words, the instructional treatment sets no specific requirement on complexity even though it is encouraged by the instructor in the whole process of its delivery.

Furthermore, learners’ changing perception of complexity might also account for its resistance to develop, as reported by the learners in the results section. It seems that intermediate EFL writers gradually shift their perception of complexity from the sentential or clausal level to the phrasal level as a result of their developing sense of written complexity. Interestingly enough, this observation is in line with the developmental prediction hypothesis (Cooper, 1976) which states that as their proficiency grows, L2 learners tend to produce writing that capitalizes on complexification at the phrasal rather than at the clausal level. For example, learners may choose to embody complex ideas in nominal or prepositional phrases rather than in embedded clauses. In other words, development in complexity manifests itself in reduced subordination rather than increased subordination. It seems that learners in the current research develop their complexity in the same way as is predicted in the hypothesis.

In addition, as was reported by most learners, they usually adopted a safety-first approach in writing tasks and thus prioritize accuracy over complexity, which is supposedly a consequence of joint production. The outcome of this approach is naturally reduced attention to complexity and ultimately its resistance to develop.

## Conclusions

This study investigated the influence of joint production on L2 writing and learners’ attitudes towards it as a pedagogy. The results were promising that joint production could effectively improve learners’ written accuracy and was perceived as a useful instructional activity by learners. Besides, it was also observed that contrary to common assumptions, aspects of writing did not develop simultaneously and linearly. The developmental changes were characterized by constantly improving accuracy and stabilizing complexity.

These findings provide useful insights into the value of joint production as an instructional activity and the developmental changes of writing among intermediate classroom learners. A number of implications can be drawn for both L2 writing research and teaching. Theoretically, it offers further evidence for the positive roles of WCF and collaborative writing in L2 writing, lending support to the social constructivist understanding of learning and cognitive development. Feedback, from the perspectives of SCT, is a purposeful activity to engage students in meaningful interactions under the guidance of a more capable “expert”. When provided within learners’ ZPD, this assistance is likely to facilitate learning. Besides, the effectiveness of feedback is largely determined by how it is delivered. To encapsulate, effective feedback should be situated in meaningful activities, contingent on learners’ needs, and moderate in amount. Similarly, it improves our understanding of second language writing development in the instructional EFL context that it is a long, nonlinear process (Caspi, 2010), particularly with syntactic complexity.

Pedagogically, the current study provides an instructional option for L2 writing teachers, particularly in the foreign language context where there is a poverty of stimulus for learners. However, to successfully implement this pedagogy, the following points should be considered. As reading materials may be necessary to the emergence of collaborative dialogue (Yang, 2016), teachers should pay due attention to the selection of materials which we believe, should cater to learners’ language proficiency levels and interest. Secondly, students need training before they can be well adapted to this in-class activity. In some cultures where students usually remain passive learners in the classroom, it is advisable to establish a student-friendly atmosphere which encourages participation. Thirdly, teachers should take flexible roles at different stage of joint production. They can be classroom managers at the initial stage, observers, language guides, facilitators, and participants at the ensuing stages.

Similarly, the findings also have some implications for feedback delivery in class. Generally speaking, immediacy should be primary condition for the feedback to be registered in learners’ mind. In other words, immediately delivered feedback easily attracts learners’ attention and is more likely to be internalized. Therefore, this principle, or the “here-and-now principle” in task-based language teaching (Robinson, 2011) should be given the top priority to. Besides, the finding of the study suggests that cross-modally delivered feedback seems to have a better effect. Therefore, L2 writing teachers should adopt a flexible approach towards learners’ mistakes, whether they appear in speech or in writing.

The current study has its own strengths. It is classroom-based and thus ecologically valid. In other words, the writing samples were collected not just for research purposes, but also as part of the curriculum requirements. Therefore, the data would be reasonably more reliable and valid than those elicited in laboratory settings. In addition, we also manipulated the genre of writing and thus eliminated any possible influence of genres on writing quality. Instead of the advantages, the study is not without limitations. It might be constrained by such factors as sample sizes, length of the study, learners’ language proficiency and the choice of linguistic measures. Therefore, the research findings should be interpreted and generalized discreetly.

Further longitudinal classroom-based research is in need to investigate the effects of joint production on other aspects of second language writing among learners of different language proficiencies. For example, whether the instruction can result in improved lexical complexity is an interesting question worth studying. Besides, as the current study suggests that L2 writing development might be non-linear and individual-specific, more case studies within the dynamic systems theory framework should be done in order to unveil the dynamics of L2 writing over time.

## Appendix 1: Interview questions

1. What difficulties did you have in English writing? Can you give me some examples?
2. Have you found progress after a semester? If yes, in what aspects?
3. Are you satisfied with your language quality in writing? Why or why not?
4. Do you pay attention to your language accuracy or complexity? For what reasons?
5. What do you think of the synopsis writing task in class? Is it useful or challenging? Why?
6. What do you find lacking in your English writing ability? How to bridge the gap?
7. What do you think of the instructor’s role in the synopsis writing task? Does it have any influence on your writing?
8. What do you usually do in the task? Does it help improve your writing? In what ways?
9. Does it help to read the 14 pieces of writing? In what way?
10. What strategies do you use in English writing?
